# A Case of Beta-Propeller Protein-Associated Neurodegeneration With a Unique Truncating Variant in the WDR45 Gene and Uncommon Clinical and Radiologic Findings

**DOI:** 10.7759/cureus.58127

**Published:** 2024-04-12

**Authors:** Simon Esbit, Richard Sidlow

**Affiliations:** 1 Medicine, Medical School for International Health, Ben Gurion University of the Negev, Be'er Sheva, ISR; 2 Medical Genetics and Metabolism, Valley Children's Hospital, Madera, USA

**Keywords:** genotype-phenotype correlations, wdr45, pediatric genetics, pediatric rare diseases, beta-propeller protein-associated neurodegeneration (bpan), neurodegeneration with brain iron accumulation (nbia)

## Abstract

Beta-propeller protein-associated neurodegeneration (BPAN), a subtype of neurodegeneration with brain iron accumulation, is caused by variants in the WDR45 gene. In this paper, we describe a patient with an atypical presentation of BPAN whose whole exome sequencing revealed a previously unattested truncating variant in the WDR45 gene (c.830+3G>C/p.Leu278Ter), the pathogenicity of which was verified by RNA transcriptomics. A number of uncommon neuroanatomic and clinical findings in our patient are discussed, expanding the phenotype associated with BPAN. This unique case challenges existing genotype-phenotype correlations and highlights the role of X chromosome skewing in shaping the clinical spectrum of BPAN.

## Introduction

Beta-propeller protein-associated neurodegeneration (BPAN) (OMIM# 300894) is one of 12 subtypes of neurodegeneration with brain iron accumulation (NBIA), encompassing a clinically and genetically heterogeneous group of diseases characterized by abnormal iron deposition in the basal ganglia accompanied by progressive extrapyramidal findings [1,2]. NBIA disorders are classified as ultra-rare diseases, with BPAN having an overall prevalence of 2-3 cases per one million people [3]. Historically, BPAN was referred to as static encephalopathy of childhood with neurodegeneration in adulthood [4].

BPAN is caused by variants in the WDR45 gene localized at Xp11.23 which codes for a beta-propeller protein, WD repeat domain phosphoinositide-interacting protein 4 (WIPI-4) [5]. Beta propeller proteins are often composed of WD-repeats, each typically consisting of a core unit of approximately 40 amino acids, ending with tryptophan (W) and aspartic acid (D) residues. Proteins containing WD-repeats are involved in a variety of functions, including apoptosis, autophagy, signal transduction, and transcription regulation [6]. The deficiency of WIPI-4 observed in BPAN is hypothesized to impair autophagy, ferritinophagy, and ferroptosis [5,7,8].

BPAN has a strong predilection for females over males as hemizygosity is typically lethal [6,9]. The majority of WDR45 mutations arise *de novo* from both germline and somatic mutations with most cases of BPAN in males attributed to postzygotic somatic mutations. Skewed X chromosome inactivation and the stage of development at the time of mutation impact the phenotypic severity of BPAN in females [6]. Hemizygous males exhibit more pronounced phenotypes; however, males with somatic mosaicism tend to have milder phenotypes than their heterozygous female counterparts [9].

The clinical presentation of BPAN changes over time. In childhood, BPAN may present as delayed psychomotor development, intellectual disabilities, epilepsy, and spasticity. During adulthood, BPAN may manifest as progressive dystonia, parkinsonism, ocular defects, sleep perturbation, and dementia [5,6,10]. In addition to cerebral and cerebellar atrophy, BPAN has a unique neuroradiologic appearance on magnetic resonance imaging (MRI) due to iron deposition: hypointense T2 signals in the substantia nigra (SN) and globus pallidus, or hyperintense halo T1 signals in the SN and cerebral peduncles, accompanied by a hypointense central band [3,6,10,11]. Postmortem findings show iron accumulation in the SN and globus pallidus, severe neuronal loss with gliosis in the SN, and various tau-positive neurofibrillary tangles throughout the brain [10,12].

Below, we describe a case of BPAN observed in a six-year-old female caused by a previously unattested truncating variant in the WDR45 gene with uncommon clinical and neuroanatomic findings.

## Case presentation

Our patient was born at 37 weeks gestation via repeat C-section to a G4P4 32-year-old mother. Dilation of the cisterna magnum was seen on the second-trimester anatomy scan and prompted concern for Dandy-Walker malformation. The pregnancy was complicated by maternal anemia and leg cellulitis; otherwise, the unrelated parents were healthy without a family history of neurodevelopmental disorders. At birth, her weight was 2.92kg (24th %ile), length was 50.75cm (71st %ile), and head circumference (HC) was 34.25cm (62nd %ile). She had an Apgar score of 8 at one minute and 9 at five minutes. At one day of age, a non-contrast MRI of the brain visualized mega cisterna magna. She was discharged from the hospital four days after birth.

At eight months of age, her growth and development were normal. She had notable left occipital plagiocephaly causing left ear displacement anteriorly, right-sided torticollis, bilateral temporal narrowing, and her anterior fontanelle was closed. An ophthalmologic exam at this time was normal. A non-contrast computed tomography scan of her head with 3-D reconstruction revealed flattening of the posterior skull, minor bilateral frontal bossing, a premature bilateral fusion of the anterior aspect of the squamosal sutures, the absence of the anterior fontanelle, and unfused coronal sutures, which appeared more diastatic than expected for her age.

**Figure 1:**
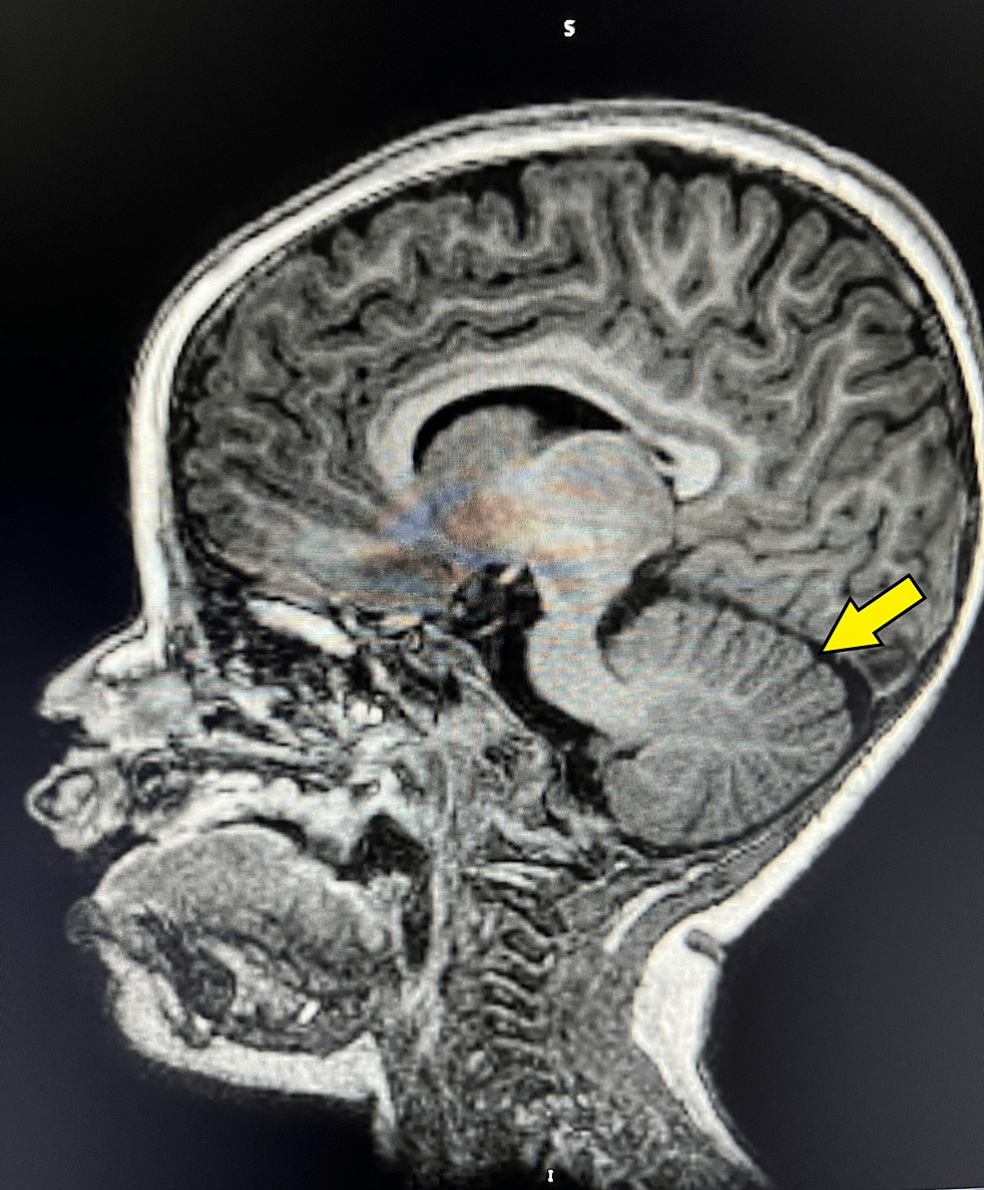
T1-weighted sagittal MRI A T1-weighted image displays a serrated pattern and cortical thickening in the peripheral cerebellar folia (yellow arrow).

**Figure 2:**
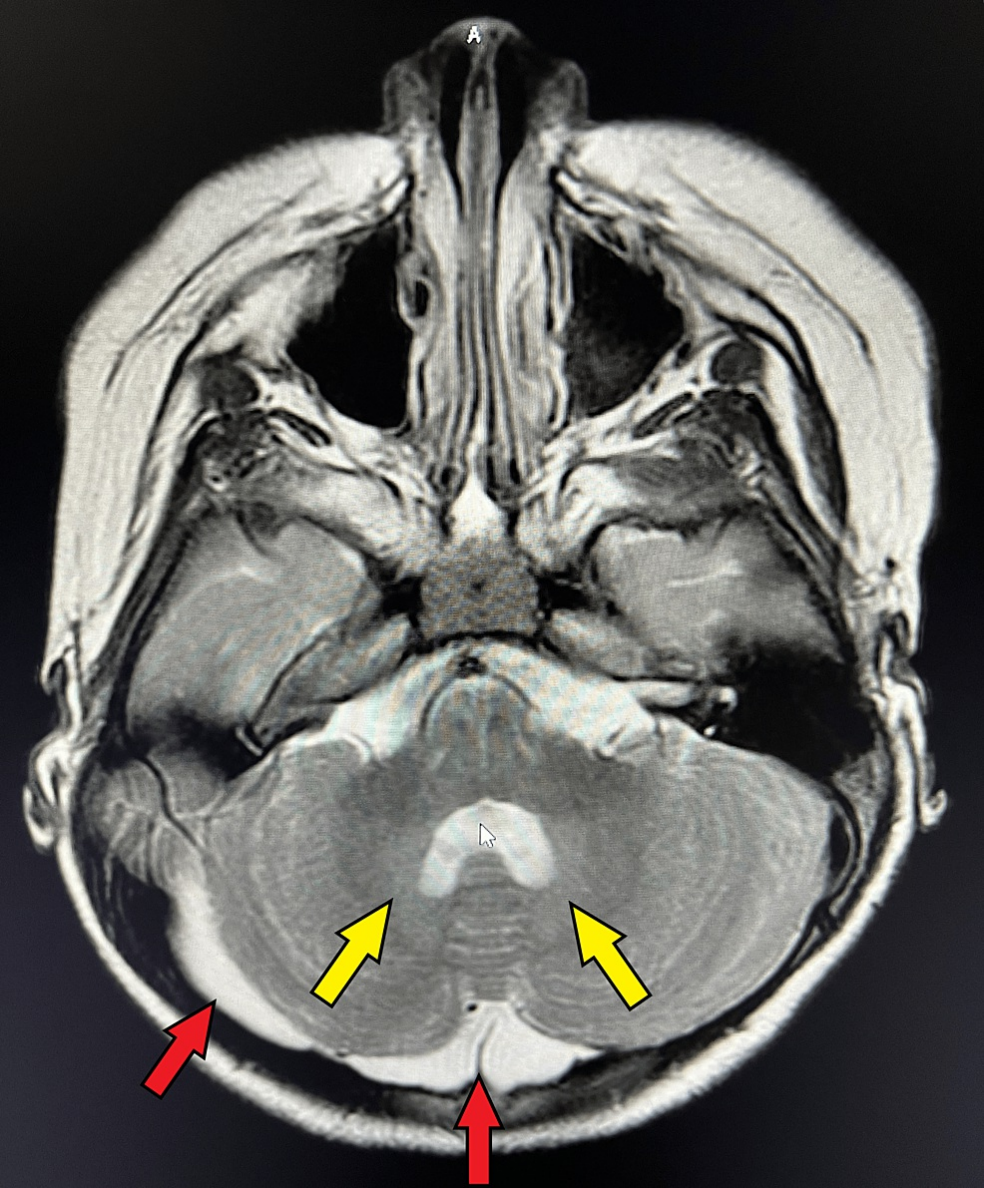
T2-weighted axial MRI A T2-weighted image displays mild cerebellar dysplasia along with a notable signal within the dentate nuclei (yellow arrows). Additionally, collections of cerebral spinal fluid are visible peripheral to the right cerebellar hemisphere and posterior to the cerebellar vermis (red arrows). These may be arachnoid cysts.

Our patient first underwent genetic consultation at 2.5 years of age. Global developmental delay was noted. She was non-ambulatory and had minimal comprehension of basic commands. She weighed 23.6kg (>99th %ile), her height was 82.7cm (4th %ile), had a BMI of 34.5 (>99th %ile), and had an HC of 47cm (24th %ile). Physical examination revealed a 1cm diameter region of cutis aplasia in the occipital region of the scalp with minimal scarring. At this time, a T1-weighted sagittal MRI of the brain revealed a cerebellum with a serrated appearance and cortical thickening in multiple folia (Figure 1). A T2-weighted axial MRI revealed mild cerebellar dysplasia and collections of cerebral spinal fluid peripheral to the right cerebellar hemisphere and posterior to the cerebellar vermis, raising suspicion for arachnoid cysts. Additionally, prominent signals were observed in the dentate nuclei (Figure 2). No genetic testing was performed at this time.

The patient returned for a genetics follow-up at age six. Additional findings were noted on physical examination: brachycephaly, upslanting eyebrows, mild hypotelorism, prominent ears, downslanting corners of the mouth, midfacial hypoplasia, and bilateral hypoplasia of the fourth toe. She weighed 21.8kg (63rd %ile) and had a HC of 50cm (29th %ile). After gaining informed consent for genetic testing, whole exome trio sequencing was performed (GeneDx, Gaithersburg, USA) which revealed a heterozygous *de novo* likely pathogenic variant (c.830+3 G>C/p.Leu278Ter) in intron 10 of the WDR45 gene (NM_007075.3) (chrX: 48933020). This was followed by single-gene RNA transcriptomics (Praxis, Atlanta, USA) demonstrating that approximately 10% of transcripts were correctly spliced, predicting a drastic reduction in full-length protein production.

## Discussion

A literature review of BPAN patients conducted by Stige et al. in 2018 showed that 91.5% of patients had *de novo* mutations with females representing over 85% of the cases [11]. Those with mild phenotypes were attributed to favorable X chromosome skewing or somatic mosaicism. Like all previously documented BPAN patients, our patient presented with developmental delay, both intellectual and psychomotor. Although various dysmorphic features were noted in 68.8% of the cases reported by Stige et al. in 2018, no unifying phenotype was identified. Our patient presented with two findings that to date have not been documented previously in BPAN patients, namely childhood history of aplasia cutis congenita and bilateral hypoplasia of the fourth toe. Unlike many of the cases analyzed by Stige et al. in 2018, our patient did not have a history of Rett-like features nor infantile-onset epileptic encephalopathy which was found in 28% and 67.7% of cases, respectively. Furthermore, our patient did not display MRI findings consistent with the typical presentation of BPAN. Ninety percent of the patients in Stige’s literature review had MRI findings consistent with iron deposition in the basal ganglia, pathognomonic for BPAN. Stige et al. also noted cerebellar and cerebral atrophy in 27% and 69.80% of their patients, respectively. Our patient manifested no brain atrophy but rather had a history of mega cisterna magna, possible arachnoid cysts, and mild cerebellar dysplasia.

Too few cases of BPAN to date have been documented for strong genotype-phenotype correlations to be made. Of note, the presence of increased iron deposition on MRI is not predictive of the clinical presentation nor trajectory of BPAN. Moreover, it is possible that the pathognomonic halo of T1 hyperintensity around a hypointense band in the cerebral peduncles may be a radiologic finding that occurs outside of the youngest pediatric age group [3]. While the variant in our patient was germline, variations in X chromosome skewing in different parts of the body may underlie the nature and severity of her findings.

Currently, there is no cure for BPAN and treatment is primarily focused on symptom management. Treatment options depend on the specific symptoms and severity of the disease. Pharmacological treatments, such as antiepileptic drugs, benzodiazepines, and melatonin, can help control seizures and improve sleep disturbances. Physical therapy can help motor function and mobility, while speech and occupational therapy can improve communication and other functional impairments [3]. Further research is needed to identify novel therapeutic strategies that target the underlying mechanisms of BPAN and improve the prognosis for affected individuals. Additionally, there are several support groups available to assist individuals and families affected by BPAN, including the BPAN Warriors (https://www.bpanwarriors.org/) and the NBIA Disorders Association (https://www.nbiadisorders.org/about-nbia/bpan), which provide valuable resources and support for those impacted by this condition.

## Conclusions

We present a patient with BPAN due to a previously unattested truncating variant heterozygous *de novo* likely pathogenic variant (c.830+3 G>C/p.Leu278Ter) in intron 10 of the WDR45 gene (NM_007075.3) (chrX: 48933020). RNA transcriptomics was utilized to confirm the pathogenic splicing effect this variant imparted on this gene. Our patient presented with several uncommon neuroanatomical features, including a history of mega cisterna magna, possible arachnoid cysts, and mild cerebellar dysplasia, as well as the clinical features of aplasia cutis in childhood and bilateral hypoplasia of the fourth toe. These findings broaden the known phenotype associated with BPAN and underscore the possible organ-specific influence of X chromosome skewing on shaping the clinical spectrum of BPAN.
